# TDP-43 regulates GAD1 mRNA splicing and GABA signaling in *Drosophila* CNS

**DOI:** 10.1038/s41598-021-98241-z

**Published:** 2021-09-21

**Authors:** Giulia Romano, Nikola Holodkov, Raffaella Klima, Fabian Feiguin

**Affiliations:** 1 International Centre for Genetic Engineering and Biotechnology (ICGEB), Padriciano 99, 34149 Trieste, Italy; 2 Department of Life and Environmental Sciences, University of Cagliari, 09042 Monserrato, Cagliari Italy

**Keywords:** Diseases of the nervous system, Genetics of the nervous system

## Abstract

Alterations in the function of the RNA-binding protein TDP-43 are largely associated with the pathogenesis of amyotrophic lateral sclerosis (ALS), a devastating disease of the human motor system that leads to motoneurons degeneration and reduced life expectancy by molecular mechanisms not well known. In our previous work, we found that the expression levels of the glutamic acid decarboxylase enzyme (GAD1), responsible for converting glutamate to γ-aminobutyric acid (GABA), were downregulated in TBPH-null flies and motoneurons derived from ALS patients carrying mutations in TDP-43, suggesting that defects in the regulation of GAD1 may lead to neurodegeneration by affecting neurotransmitter balance. In this study, we observed that TBPH was required for the regulation of GAD1 pre-mRNA splicing and the levels of GABA in the *Drosophila* central nervous system (CNS). Interestingly, we discovered that pharmacological treatments aimed to potentiate GABA neurotransmission were able to revert locomotion deficiencies in TBPH-minus flies, revealing novel mechanisms and therapeutic strategies in ALS.

## Introduction

A common characteristic shared by several neurodegenerative diseases is the dysfunction of the RNA-binding protein TDP-43, a member of the heterogenous nuclear ribonucleoproteins (hnRNPs) family ^1,2^. TDP-43 is a protein involved in several aspects of RNA metabolism including pre-mRNA splicing, mRNA transport and micro RNA maturation ^3–6^. A breakthrough link with neurodegenerative diseases came in 2006, when TDP-43 was identified as the main component of the ubiquitinated cytoplasmic inclusions in ALS and frontotemporal lobar degeneration (FTLD) ^7–9^. TDP-43 pathology is currently described in a large proportion of cases of Alzheimer’s disease as well as Parkinson’s and Huntington’s disease ^10–14^. Attentive studies aimed to understand the normal function of TDP-43 and its participation in the mechanisms of neurodegeneration have, therefore, became critical to establish the metabolic pathways implicated in TDP-43-mediated neuronal toxicity. In this direction we have previously indicated that TDP-43 is required to regulate the synaptic levels of GAD67, the enzyme responsible for converting glutamate to γ-aminobutyric acid (GABA), suggesting that modifications in the glutamate/GABA neurotransmitter balance may affect neuronal survival in TDP-43 perturbed brains ^15^. In this direction, evidences of the involvement of glutamate in the pathogenesis of ALS disease were already suggested by several studies ^16–18^. However, treatments aimed to prevent glutamate mediated excitotoxicity have failed to correct the clinical symptoms of the disease, revealing that different mechanisms might be at work besides the excessive availability of glutamate. In support of this hypothesis, it has been reported that GABA levels are reduced in motor cortex of patients with ALS and demonstrated that TDP-43 overexpression increased GAD67 protein levels and GABA release in mouse forebrain ^19–22^, implying that the synaptic transmission mediated by GABA might be affected or/and may have a role in TDP-43 defective brains. In this study we investigated the mechanisms by which TDP-43 regulates the cytoplasmic levels of GAD1 and determined the role of GABA neurotransmission in TDP-43 pathology using *Drosophila*.

## Results

### *Drosophila* TDP-43 regulates the expression levels of GAD1 by facilitating its pre-mRNA splicing

**Figure 1:**
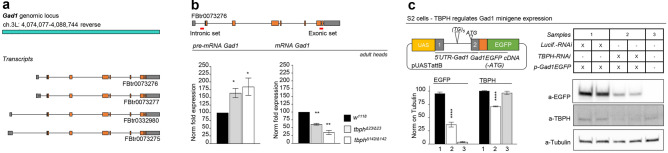
TBPH modulates GAD1 protein expression by regulating its pre-mRNA 5’-UTR splicing. **(a)** Scheme of Gad1 genomic locus and transcripts in *Drosophila*. (**b)** qRT-PCR of Gad1 pre-mRNA left panel and mRNA right panel on adult heads of w^1118^, tbph^Δ23/Δ23^ and tbph^Δ142/Δ142^. *n* = 3; *p < 0.05 and **p < 0.01 calculated by one-way ANOVA. (**c)** Western blot analysis for GAD1 minigene expression in S2 cells (anti-EGFP, anti-TBPH, and anti-Tubulin). Full-length gels in Supplementary Information. Sample 1: S2 cells co-transfected with GAD1 minigene, Actin-GAL4 and Luciferase-RNAi. Sample 2: S2 cells co-transfected with GAD1 minigene, Actin-GAL4, and TBPH-RNAi. Sample 3: S2 cells only. In the graph the quantification of EGFP and TBPH expression weighted on Tubulin of Luciferase-RNAi (black column) compared with TBPH-RNAi (white columns), in light gray S2 cells only as negative control. ****p < 0.0001; calculated by one-way ANOVA. In the scheme GAD1 minigene: the 5’ UTR region of Gad1 spanning from exon1 to exon2 in frame with a reporter EGFP has been cloned in pUAST attB plasmid. *n* = 2, Error bars SEM.

We have previously demonstrated that the protein levels of GAD1 appeared downregulated in TBPH-null flies, however, the mechanisms behind these modifications were not identified ^15^. In order to address this question, and considering that TBPH is involved in the regulation of different aspects of RNA metabolism, we decided to investigate whether GAD1 RNA splicing was affected in TBPH-minus alleles (two TBPH null allele strains, named Δ23 and Δ142, in which TBPH protein production is completely abolished, see ^23^). To this purpose, we designed a set of primers specific to discriminate GAD1 pre-mRNA and another one specific for mRNA (respectively the intronic set and the exonic set, (see Fig. 1a,b), and quantified their expression levels by qRT-PCR. Interestingly, we found that the pre-mRNA of GAD1 appeared upregulated in the brains of the TBPH-mutant alleles compared to controls (Fig. 1b, left graph). On the contrary, the mature mRNA transcript of GAD1 was downregulated in the mutant flies judged against controls (Fig. 1b, right graph) indicating that TBPH is required to regulate the processing of GAD1 mRNA, most probably, through the splicing of the mature transcript in *Drosophila* brains. In agreement with this possibility, we observed the presence of putative binding sites for TBPH in the long intron present at the 5’UTR region of GAD1, between the non-coding exon1 and the coding exon2 (Fig. 1c, upper scheme). In order to test if the absence of TBPH influenced the processing of these segments, we constructed a minigene containing the genomic sequences described above (Fig. 1c, upper left scheme). The GAD1 pre-mRNA 5’-UTR minigene also presented an EGFP cloned in frame with the second exon of GAD1 that carried the original ATG starting codon. The construct was placed under the control of the GAL4-UAS system and used to transfect *Drosophila* S2 cells under the Actin promoter (actin-GAL4). Thus, S2 cells co-transfected with an RNAi against TBPH showed a strong reduction in the expression of the EGFP protein compared to control cells treated with an RNAi against luciferase in western blot assays (Fig. 1c right panel, quantified in the left graph). These results show that TBPH function is required for the proper splicing and expression of the GAD1 pre-mRNA 5’-UTR minigene and strongly suggest that similar alterations may explain the defects detected in the processing of the immature GAD1 mRNA in TBPH-null flies.

### Reduced levels of GABA neurotransmitter in TDP-43-null brains affect locomotor behaviors

The alterations in the processing of GAD1 immature mRNA described above insinuate that GABA neurotransmission might be affected in TBPH-null flies. In fact, the synthesis of GABA occurs through the conversion of glutamate by GAD1 enzyme; then GABA is loaded into vesicles by the vesicular GABA transporter (vGAT) ^24^. GABA receptors are inhibitory receptors that respond after GABA released. The GABA receptors are divided into two types: the ionotropic and the metabotropic. To date in *Drosophila* three ionotropic subunits and three metabotropic subunits have been described (see Table 1). In order to address this hypothesis, we decided to test whether modifications in GABA signaling were able to influence the locomotor phenotypes described in TBPH deficient flies. For these experiments, we took advantage of the GAL4-UAS expression system to simultaneously co-silence the endogenous TBPH protein together with the various GABA ionotropic and metabotropic receptors.

**Table 1:** GABA receptors and transporters.

*Drosophila* GABA receptor/transporter	Gene CG	Classification	RNAi fly	Human ortholog	Reference
RDL (resistant to dieldrin)	10537	GABA_A_ Ionotropic	#41101 (VDRC) #100429 (VDRC)	GABRP	^39,40^
LCCH3 (ligand-gated chloride channel homolog 3)	17336	GABA_A_ Ionotropic	#37409 (VDRC) #109606 (VDRC)	GABRB1	^41,42^
GRD (gaba receptor/glycine receptor)	7446	GABA_A_ Ionotropic	#38384 (VDRC) #58175 (VDRC)	GABRA4	^42,43^
GABA-B-R1 (gaba-b receptor subtype 1)	15274	GABA_B_ Metabotropic	#101440 (VDRC)	GABBR1	^44^
GABA-B-R2 (gaba-b receptor subtype 2)	6706	GABA_B_ Metabotropic	#1784 (VDRC) #1785 (VDRC) #110268 (VDRC)	GABBR2	^44^
GABA-B-R3 (gaba-b receptor subtype 3)	3022	GABA_B_ Metabotropic	#50622 (VDRC) #108036 (VDRC)	GABBR2	^44^
Vgat (vesicular gaba transporter)	8394	Vesicular transporter	#45916 (VDRC)	SLC32A1	^45,46^

**Figure 2:**
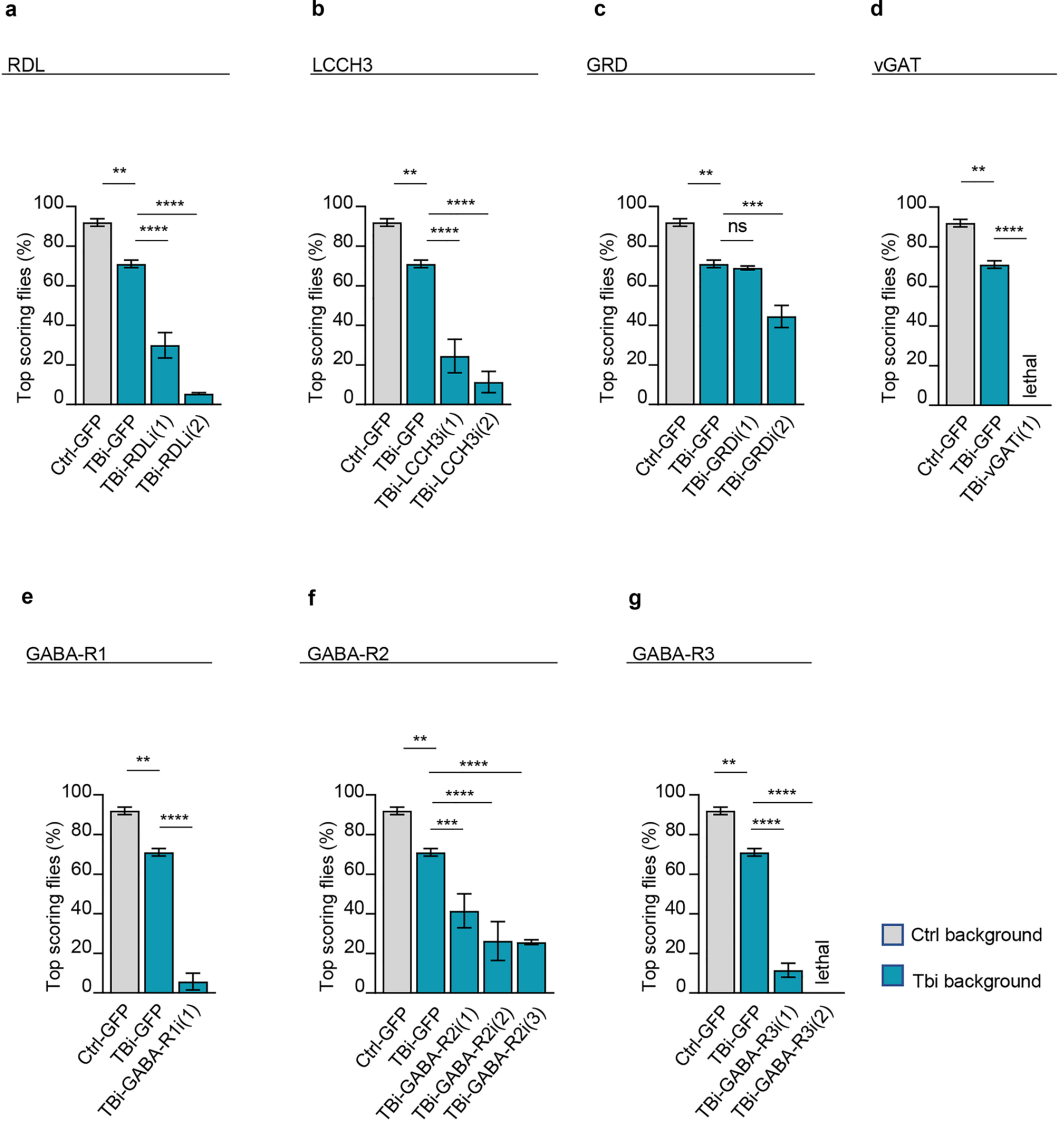
Silencing of GABA receptors and transporter worsen the TBPH hypomorphic phenotype. **(a–g)** Climbing assay of 4 days adult flies of control (Ctrl-GFP = tbph^Δ23^,*elav*-GAL4/UAS-GFP;UAS-Dicer/ +), TBPH hypomorphic (TBi-GFP = tbph^Δ23^,*elav*-GAL4/UAS-GFP;UAS-Dicer/UAS-TBPH-RNAi) and TBPH hypomorphic with several GABA receptors silenced (TBi-GABA receptors name = tbph^Δ23^,*elav*-GAL4/UAS-GABA-receptor RNAi;UAS-Dicer/UAS-TBPH-RNAi). In (**a)** RDL-RNAi line (1)   #41101, line (2) #100429; in (**b)** LCCH3-RNAi line (1) #37409, line (2) #109606; in (**c)** GRD-RNAi line (1) #38384, line (2) #58175; in (**d)** vGAT-RNAi line (1) #45916; in (**e)** GABA_B_ receptor type 1 line (1) #101440; in (**f)** GABA_B_ receptor type 2 line (1) #1784, line (2) #1785, line (3) #110268; in (**g)** GABA_B_ receptor type 3 line (1) #50622, line (2) #108036. The total number of tested animals per genotype was *n* > 50. *ns* not significant, *p < 0.05, **p < 0.01, ***p < 0.001 and ****p < 0.0001; calculated by one-way ANOVA. Error bars SEM.

Thus, specific RNAi against the GABA_A_ type receptor (RDL), the ligand-gated chloride channel (LCCH3), the GABA_B_ type receptor 1, 2 and 3 (R1, R2 and R3) were expressed, either alone or together with an RNAi against TBPH, under the neuronal driver *elav*-GAL4. As a result, we found that the suppression of the GABA receptors strongly enhanced the motility problems present in TBPH-defective flies compared to control insects expressing the RNAi against the GABA receptors alone (Fig. 2 and Supplementary Fig. 1).

**Figure 3:**
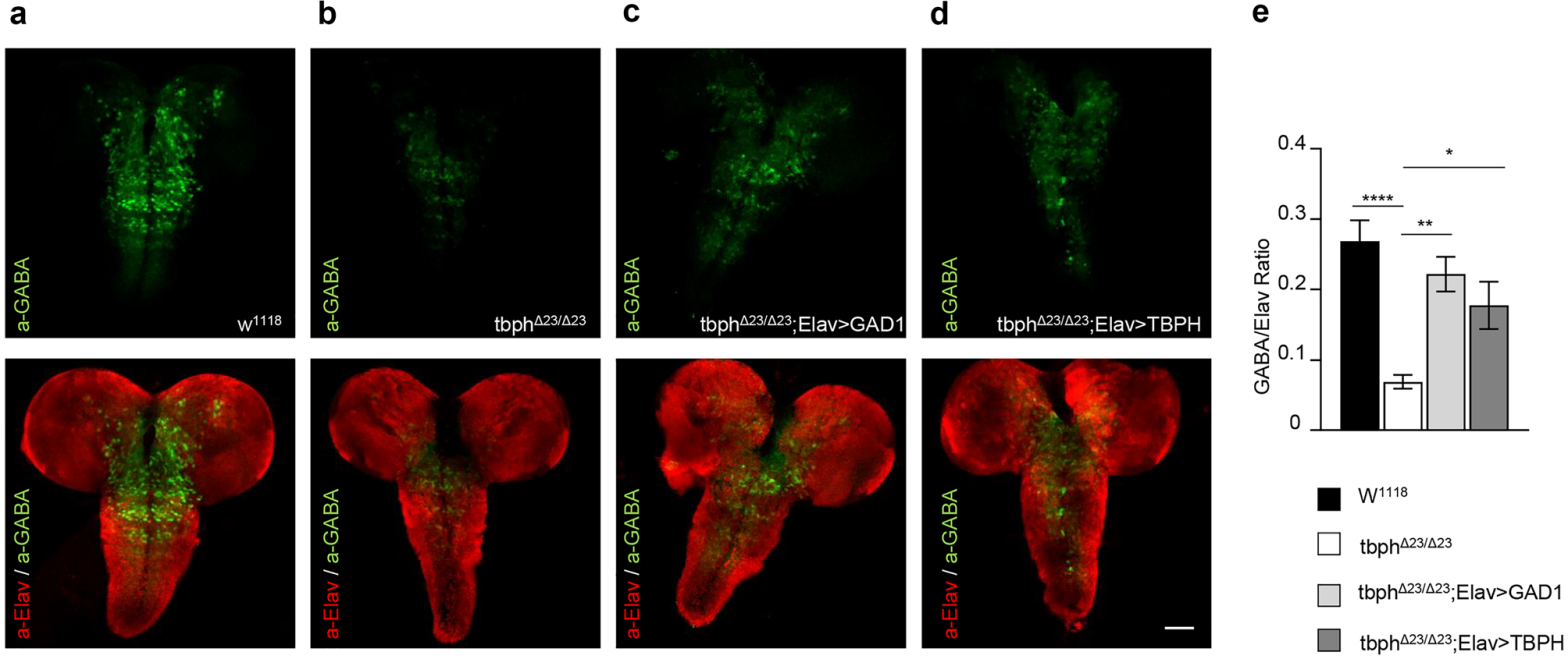
GABA was strongly downregulated in TBPH minus larval brain and recovered by GAD1 and TBPH expression in neurons. **(a–d)** Confocal microscopy acquisition of whole larval brain. GABA neurotransmitter was labeled with anti-GABA (Green) and neurons were labeled anti-Elav (Red, merged to GABA) of w^1118^ (**a**), tbph^Δ23/Δ23^ (**b**), tbph^Δ23^,*elav*-GAL4/tbph^Δ23^;UAS-GAD1/ + (**c**), and tbph^Δ23^,*elav*-GAL4/ tbph^Δ23^, UAS-TBPH (**d**). (**e)** Quantification of whole brain intensity GABA/Elav ratio. The number of examined brains per genotype was *n* > 10. *p < 0.05, **p < 0.01, ****p < 0.0001 calculated by one-way ANOVA. Error bars SEM. Scale bar: 50 µm.

In the same direction, we utilized a specific antibody against GABA to quantify the intracellular levels of the neurotransmitter in third instar larval brain. After the staining, we found that the levels of GABA intensity appeared significantly reduced in TDP-43-null brains compared to wild type controls (Fig. 3a,b,e). Interestingly, we found that the neuronal transgenic expression of GAD1 or the endogenous protein TBPH in TDP-43-minus backgrounds were able to recover the expression levels of GABA in *Drosophila* brain (Fig. 3c,d,e), indicating that these results are specific and the regulation of GAD1 levels is critical to prevent GABA neurotransmission defects and neurodegeneration in TDP-43-defective brains.

### The recovery of GABA neurotransmission improves locomotion in TDP-43-null flies

**Figure 4:**
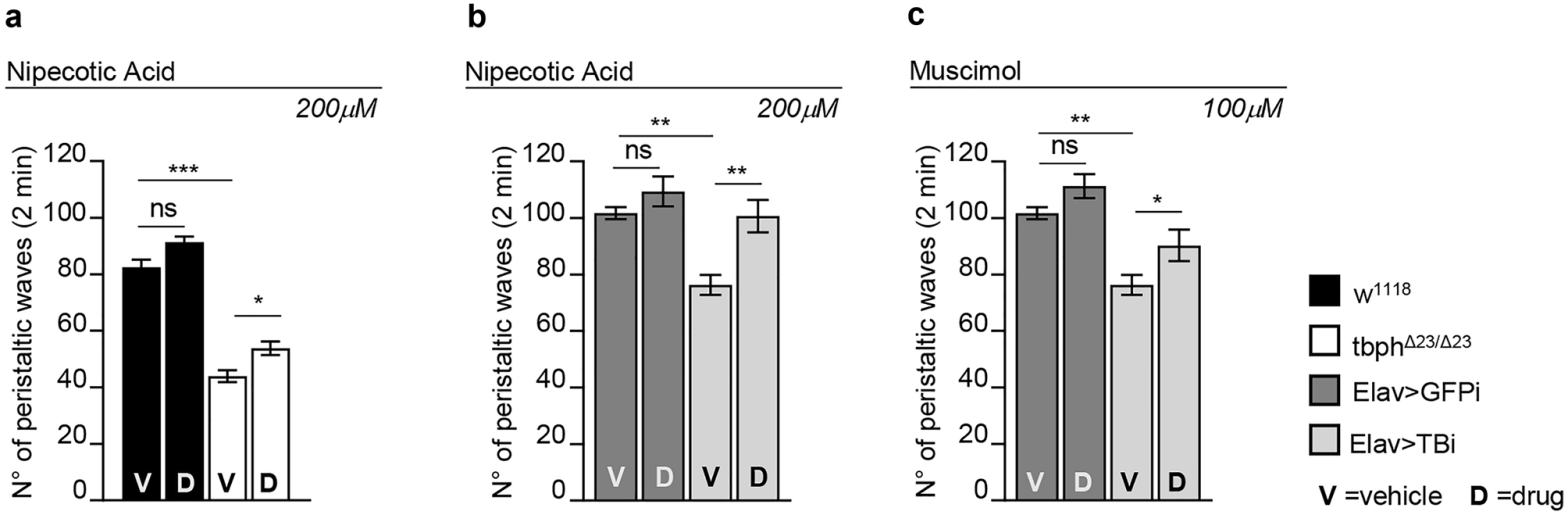
Pharmacological GABA stimulation ameliorates the motility defects in TBPH dysfunction. **(a)** Peristaltic larval waves of third instar larvae of w^1118^ (black columns) and tbph^Δ23/Δ23^ (white columns) fed with 200 µM of Nipecotic Acid (D) and vehicle only (V). (**b)** Peristaltic larval waves of third instar larvae of Elav > GFPi (w,UAS-Dicer/ + ; tbph^Δ23^,*elav*-GAL4/UAS-GFP-RNAi) (dark gray columns) and Elav > TBi (w,UAS-Dicer/ + ; tbph^Δ23^,*elav*-GAL4/ + ;UAS-TBPH-RNAi/ +) (light gray columns) fed with 200 µM of Nipecotic Acid (D) and vehicle only (V). (**c)** Peristaltic larval waves of third instar larvae of Elav > GFPi (w,UAS-Dicer/ + ; tbph^Δ23^,*elav*-GAL4/UAS-GFP-RNAi) (dark gray columns) and Elav > TBi (w,UAS-Dicer/ + ; tbph^Δ23^,*elav*-GAL4/ + ;UAS-TBPH-RNAi/ +) (light gray columns) fed with 100 µM of Muscimol (D) and vehicle only (V). n = 20, ns = not significant, *p < 0.05, **p < 0.01, ***p < 0.001, calculated by two-way ANOVA. Error bars SEM.

In order to determine if the reduced levels of GABA detected in TBPH-minus brains were related with the neurodegenerative phenotypes described in these flies, we decided to treat TBPH-null flies with different agonists of GABA neurotransmission. For these assays, a potent GABA uptake inhibitor, Nipecotic acid (200 µM) was added to the fly food during larvae development and we found that the administration of this compound was able to consistently improve the peristaltic movements of the TBPH-minus third-instar larvae (L3) compared to untreated mutants or wild-type controls (Fig. 4a).

The positive effect of this pharmacological treatment became more obvious when TBPH hypomorphic background was utilized. As a matter of fact, we found that 200 µM of Nipecotic Acid dispensed to flies expressing TBPH-RNAi in neurons (UAS-Dcr-2/ + ; tbph^Δ23^, *elav*-GAL4/ + ; TBPH-RNAi/ +) were sufficient to recover motility (Fig. 4b), indicating that GABA neurotransmission plays an important role in TBPH-mediated neurodegeneration. We employed a similar approach utilizing a different drug, the Muscimol (100 µM) (a known GABA_A_ receptor agonist), this showed a significative rescue of L3 larvae motility in TBPH hypomorphic background-null alleles compared to controls (Fig. 4c).

## Discussion

Defect in the neuronal handle of the excessive formation of glutamate, has been long implicated in the mechanisms of neurotoxicity behind ALS. However, growing evidences suggest that the glutamate-induced excitotoxicity hypothesis, alone, is not sufficient to explain the hyperexcitability of the motoneurons observed in ALS. In effect, one of the few approved compounds to treat ALS, riluzole, besides its anti-glutamate action directly interact with GABA receptors, signifying that the activation of inhibitory neurotransmission may also play a role in the disease. In this direction, a reduced GABA levels in the motor cortex of ALS patients were previously describe ^19,20^. Further evidences came also from SOD1 (G93A) mouse model ^25^, in which the glutamatergic neuronal hyperexcitability was associated to a decreased GABAergic intracortical inhibition ^22,26^. On a different ALS mouse model, the wobbler mice ^27^, similar evidences were described including a reduction in vesicular GABA transport followed by a decreased GABAergic inhibition ^28^. Suitably, alterations in GABA neurotransmission were described in patients suffering from Alzheimer’s disease ^29^, Parkinson’s disease ^30^ and related neurodegenerative process in addition to ALS ^20,31^. Nevertheless, despite the evidences indicated above, the role of GABA and others inhibitory neurotransmitters balance in TDP-43 pathology was not completely clarified. In that respect, our data revealed that TBPH, the TDP-43 conserved protein in *Drosophila*, plays an important role in maintaining the equilibrium between neuroexcitatory and inhibitory signals through the regulation of GAD1, the enzyme responsible for the synthesis of GABA after the cleavage and processing of glutamate. On that point, we found that TBPH has an important role in the modulation of GAD1 mRNA splicing. Thus, we observed that a reduction of TBPH promoted the accumulation of GAD1 pre-mRNA transcripts with the subsequent reduction in the processing and generation of mature GAD1 mRNA and protein. Regarding the molecular mechanisms, notwithstanding the presence of putative binding sites for TBPH in the 5’UTR region of GAD1 mRNA, we did not find a direct binding between TBPH and GAD1 mRNA ^15^. Successively, we observed that the overexpression of TBPH was not sufficient to modify the pre-mRNA processing of GAD1 either (Supplementary Fig. 2), suggesting that TBPH may act in consonance with other RNA-binding proteins in the formation of GAD1 mRNA splicing complexes ^32,33^. Regarding the physiological consequences of GAD1 reduction in TBPH/TDP-43-minus flies, we have described that the genetic correction of GAD1 levels was sufficient to restore locomotive behaviors and neurotransmitter balance in vivo ^15^, supporting the idea that defects in inhibitory signals additionally contribute to motoneurons degeneration. Moreover, our results provide molecular explanations about the mechanisms that may derive from defects in TBPH/TDP-43 to alterations in neurotransmitters balance in ALS patients. In relation with that, we exhaustively analyzed the role of each GABA receptors encoded in the *Drosophila* genome, in terms of their capacity to influence the neurological phenotypes described in TBPH/TDP-43-depleted flies. Therefore, we observed that the silencing of GABAergic receptors provoked a predominant worsening of the locomotory phenotypes in TBPH-RNAi treated larvae (Fig. 2), and no major differences could be distinguished after the suppression of either both, the ionotropic and the metabotropic, GABA receptors subtypes. Although, it is well known that ionotropic and metabotropic receptors activate two different subcellular responses after GABA activation ^24^, our experimental evidences suggest that the reduction in GABA signaling (independently of the characteristic of the GABA receptors) significantly contribute to the pathological phenotypes described in TBPH loss of function alleles. In that respect, we observed that the pharmacological potentiation of GABA signaling with nipecotic acid, an inhibitor of GABA uptake, was able to significantly revert the locomotor defects observed in TBPH-null flies. Interestingly, our results are in line with other evidences obtained from different ALS models, Boussicault et al. 2020 demonstrated that GABA agonists treatment synergistically improved the phenotype in primary cultures of motoneurons derived from SOD1 G93A rat embryos ^34^. On the other hand, clinical trials using GABA agonists to treat ALS patients have not yet yielded significant results suggesting that the modulation of GABA neurotransmission alone may not be sufficient to overcome the complete symptoms of the disease ^35^. More sophisticated therapies, perhaps combining GABAergic with anti-glutamatergic approaches, might be required to re-stablish the neurotransmitter balance in TDP-43 affected brains. In support of this hypothesis, gene-based therapies using GAD1 to modulate neuronal signaling are being used to treat patients with Parkinson’s disease and similar approaches could be evaluated in patients with ALS ^36,37^.

Altogether, our results reveal a novel molecular mechanism behind TDP-43-derived pathologies and put forward novel therapeutic strategies aimed to potentiate inhibitory signaling or balancing the levels of neurotransmitters may succeed to control motoneurons hyperexcitability in ALS affected individuals.

## Materials and methods

### Fly strains

The fly genotypes used for the experiments are indicated hereafter: w^1118^—OregonR—w;tbph^Δ23^/CyO^GFP^ (null TBPH allele, ref ^23^)—w;tbph^Δ142^/CyO^GFP^ (null TBPH allele, ref ^23^)—w;*elav*-GAL4/CyO^GFP^—w,UAS-Dicer2;*elav*-GAL4/CyO^GFP^—w;siRNA-UAS-GFP (#9330, BDSC)—w;UAS-GAD1/TM3,Sb (gifted from Dr. Andreas Prokop)—w;UAS-TBPH—w;UAS-mCD8::GFP/CyO—w;UAS-TBPH-RNAi/TM6B (#38377, VDRC)—siRNA-GABA_A_ receptor, resistant to dieldrin (RDL, #41101, #100429, VDRC)—siRNA Ligand Gated Chloride Channel (LCCH3, #37409, #109606, VDRC)—siRNA Glycine like receptor (GRD, #38384, #58175)—siRNA Vesicular GABA Transporter (vGAT, #45916, VDRC)—siRNA-GABA_B_ Receptor Type 1 (R1, #101440, VDRC)—siRNA-GABA_B_ receptor Type 2 (R2, #1784, #1785, #110268, VDRC)—siRNA-GABA_B_ receptor Type 3 (R3, #50622, #108036, VDRC).

### Climbing assay

Freshly eclosed flies were transferred in new food vials and let to adapt for 3 to 4 days. A female-male ratio of 1:1 was maintained. After this period, they were moved, without anesthesia, to 15 ml glass cylinders, tapped to the bottom and let climb taking advantage of their natural drive for negative geotaxis. The number of flies that reached the top of the tube in 15 s was counted, and converted in percentage.

### Larval brain, microscope acquisition and quantification

Larvae were selected and dissected as previously described in ^38^. Briefly, for whole larval brain staining, previously selected larvae were dissected on Sylgard plates in Phosphate Buffer (PB), brains were removed and fixed 20 min in 4% formaldehyde in Phosphate Buffer with 0.3% Triton X100 (PBT), blocked in 5% Normal Goat Serum (NGS), labelled with primary and secondary antibodies and mounted on microscope slides in Slow Fade (S36936, Thermo Fisher Scientific). Brain images were acquired on 20 × at a 0.6-fold magnification on a LSM 880 Zeiss confocal microscope and then analyzed using ImageJ. Z-stacking was performed and the ratio of GABA/Elav Max Intensity was calculated. Primary antibodies concentration: anti-Elav (1:250, DSHB), anti-GABA (1:500, Sigma). Secondary antibodies concentration: Alexa Fluor 488 (1:500, Life Technologies), Alexa Fluor 555 (1:500, Life Technologies).

### Cell culture and RNA interference

S2 cells were cultured in Insect-Xpress medium (Lonza) supplemented with 10% fetal bovine serum and 1X antibiotic–antimycotic solution (#A5955, Sigma). RNA interference was achieved using HiPerfect Transfection Reagent (#301705, Qiagen) and siRNA specific for *Drosophila* TBPH (5’-GGAAGACCACAGAGGAGAGC-3’), as control siRNA for luciferase was used (5’-UAAGGCUAUGAAGAGAUAC-3’; Sigma). Immediately before transfection 2 × 10^6^ cells were seeded in 6-well plates in 1.4 ml of medium containing 10% fetal serum. 2 µg of each siRNA, were added to 91 µl of Opti-MEM I reduced serum medium (#51985-026, Thermo Fisher Scientific), incubated 5 min at room temperature and subsequently 6 µl of HiPerfect Transfection Reagent were added. The silencing procedure was performed again after 24 h. Plasmid containing the Gad1 mini-gene and GAL4 were co-transfected (0.3 µg each) at 24 h together with the second siRNA dose. Cells were analyzed after 72 h of the initial treatment.

### GAD1 minigene construction

A mini-gene containing the 5’ UTR region of Gad1 spanning from exon1 to exon2 (ref isoform FBtr0073276) in frame with a reporter EGFP has been cloned in pUAST attB plasmid. The minigene was co-transfected with p-actin-GAL4 in S2 cells. The EGFP expression was analyzed in S2 cells treated with siRNAs against TDP-43 or luciferase.

### RT-PCR

Adult heads were mechanically squeezed to proceed with RNA extraction, using RNeasy Microarray tissue kit (#73304 Qiagen) and treated with Turbo DNA-free kit (#AM1907 Ambion). Retro-transcription was performed using oligo-dT primers and SuperScript III First-Strand Synthesis (#18080093 Invitrogen). Gene specific primers were designed for amplification:

Gad1(intronic set): *5’GCCAAACTCCGCATTCCATTT3’ and 5’ACCGAAGGCCGTGGGTCCTCG3’*;

Gad1(exon/exon set): *5’TGATCCTTGAACCGGAGTGC3’ and 5’ACCATCAGCGTTCCCTTCTG3’*;

Rpl11 *5’CCATCGGTATCTATGGTCTGGA3’ and 5’CATCGTATTTCTGCTGGAACCA3’.*

The quantification was calculated according the ΔΔC_T_ equation and then normalized on control genotype.

### Western blot

S2 cells were lysed in lysis buffer (10 mM Tris, 150 mM NaCl, 5 mM EDTA, 5 mM EGTA, 10% Glycerol, 50 mM NaF, 5 mM DTT, 4 M Urea, pH 7.4, protease inhibitors (Roche, #11836170001)) and after protein quantification of lysates by Qbit (Q#33211, Invitrogen), samples were separated on NuPAGE 4–12% Bis–Tris precast gels (Thermo Fisher Scientific) and transfer to 0.22 µm nitrocellulose membranes (#NBA083C Whatman Protran). Membranes were blocked in 5% non-fat dry milk in Tris Buffered Saline with 0.1% Tween-20 (TBS-T). Primary antibodies were used with the following concentrations: anti-GFP (1:2000, Invitrogen), anti-TBPH (1:3000, homemade ^23^ and anti -tubulin DM1A (1:5000, Calbiochem). Secondary antibodies concentration: anti-rabbit HRP conjugated (1:10,000, Pierce #32460), anti-mouse HRP conjugated (1:10,000, Pierce # 32430). For detection, SuperSignal West Femto Maximum Sensitivity Substrate Kit (Pierce, #PR34095) was used. Quantification was performed by Alliance software, after acquisition with UVItec CAMBRIDGE Alliance.

### Drug treatment

Muscimol 100 µM and Nipecotic Acid 200 µM were used. According to the final concentration needed, drugs were prepared and diluted in the fresh fly food. This solution was aliquoted in fly tubes where parental flies were transferred and let lay embryos for a period of 24 h. After this period, parental flies were discarded, and embryos were let to grow to third instar larvae, used for further analysis.

### Larval movement

Third instar larvae were individually selected, cleaned with water from any food remaining and placed on 0.7% agarose plates under stereoscope vision. After 30 s of adaptation, the number of peristaltic waves performed in 2 min were counted.

### Statistical analysis

All statistical analysis was performed with GraphPad Prism 7.0 applying the one-way ANOVA test (Bonferroni post-test) and the two-way ANOVA test (Fig. 4).

## Supplementary Information

Supplementary Figures.

## Data Availability

The datasets generated during and/or analyzed during the current study are available from the corresponding author on reasonable request.
