# Supplementary material for: TDP-43 regulates GAD1 mRNA splicing and GABA signaling in Drosophila CNS
Source: Sci Rep. 2021 Sep 21;11:18761. doi: 10.1038/s41598-021-98241-z (PMC8455590; doi:10.1038/s41598-021-98241-z)
Supplement: Supplementary file 1 — Supplementary Figures. [file 41598_2021_98241_MOESM1_ESM.pdf]

Supplementary Material

TDP-43 regulates GAD1 mRNA splicing and GABA signaling in Drosophila CNS

Giulia Romano[1]*, Nikola Holodkov[1], Raffaella Klima[1] and Fabian Feiguin[1,2]*.

1. International Centre for Genetic Engineering and Biotechnology (ICGEB), Padriciano 99, 34149 Trieste, Italy.
2. Department of Life and Environmental Sciences, University of Cagliari, 09042 Monserrato (Cagliari), Italy.

*Correspondence to: giulia.romano@icgeb.org or fabian.feiguin@icgeb.org

Phone: +39-040-3757201

Fax: +39-040-226555

Supplementary Figure 1

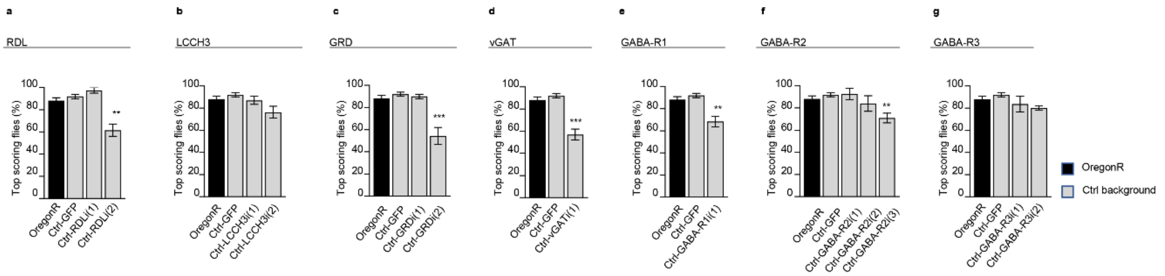

Supplementary Figure 1. Silencing of GABA receptors in wild type background. **a-g** Climbing assay of 4 days adult flies of OregonR (black column), Ctrl-GFP (tbph$^{\Delta23}$,*elav*-GAL4/UAS-GFP;UAS-Dicer/+) and Ctrl-GABA receptor name (tbph$^{\Delta23}$,*elav*-GAL4/UAS-GABA-receptor RNAi;UAS-Dicer/+). In **a** RDL-RNAi line (1)= #41101, line (2) #100429; in **b** LCCH3-RNAi line (1) #37409, line (2) #109606; in **c** GRD-RNAi line (1) #38384, line (2) #58175; in **d** vGAT-RNAi line (1) #45916; in **e** GABA$_B$ receptor type 1-RNAi line (1) #101440; in **f** GABA$_B$ receptor type 2-RNAi line (1) #1784, line (2) #1785, line (3) #110268; in **g** GABA$_B$ receptor type 3-RNAi line (1) #50622, line (2) #108036. The total number of tested animals per genotype was > 50. **$p<0.01$, ***$p < 0.001$; calculated by one-way ANOVA. Error bars SEM.

Supplementary Figure 2

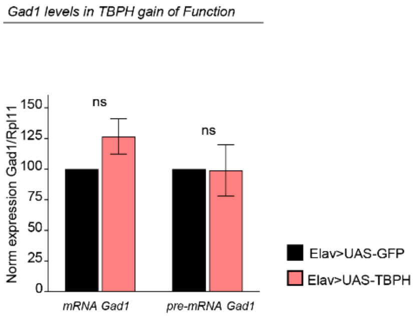

Supplementary Figure 2. GAD1 levels in TBPH gain of function larval brains. qRT-PCR of Gad1 pre-mRNA right panel and mRNA left panel on larval brains of Elav>UAS-GFP (*elav*-GAL4/UAS-GFP) and Elav>UAS-TBPH (*elav*-GAL4/UAS-TBPH). *n*=2; ns=not significant; calculated by t-test. Error bars SEM.

Supplementary Information

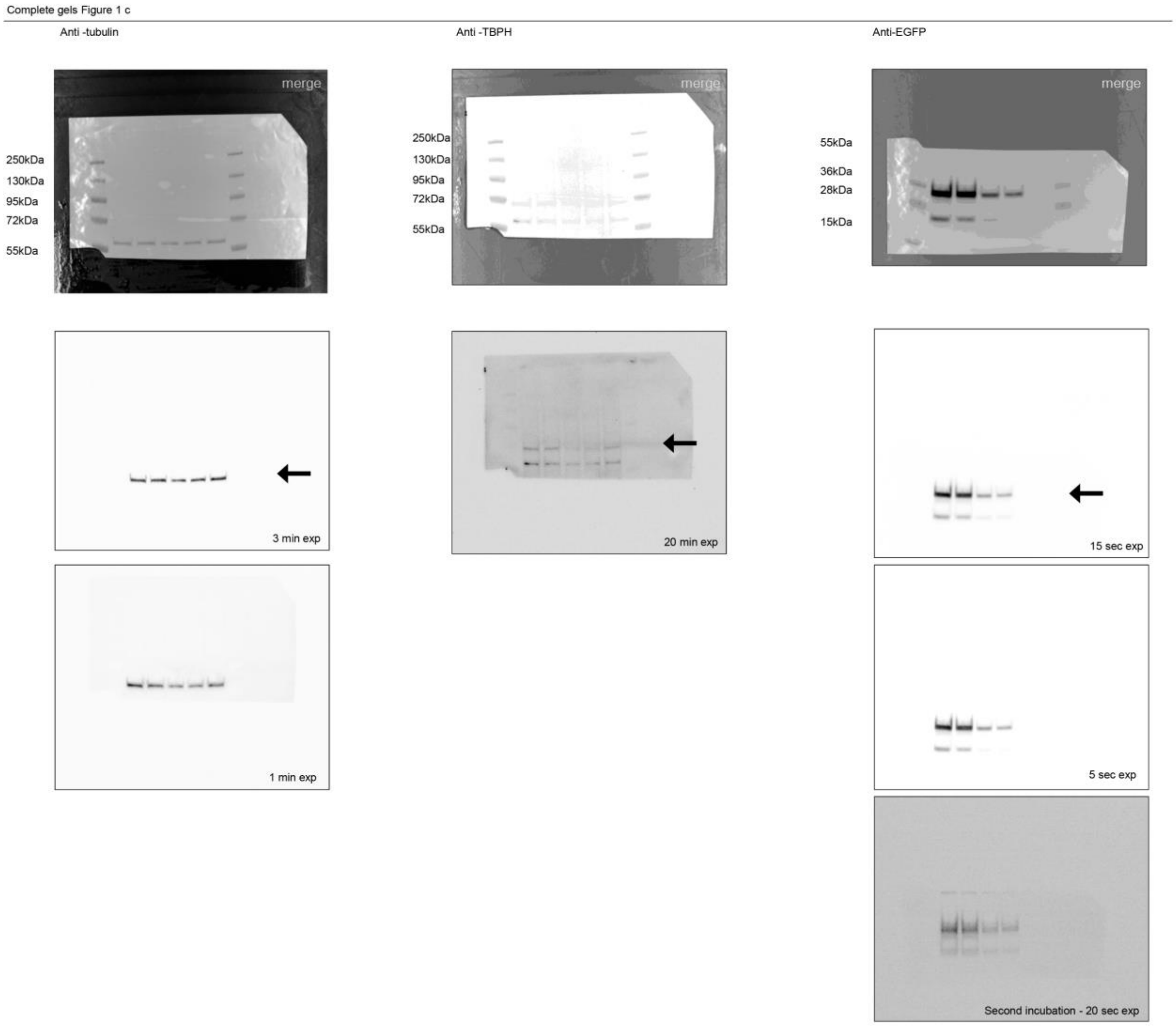

Supplementary Material. Full-length gels of Figure 1c. Western blot analysis of S2 cells probed with anti-Tubulin (left panel), anti-TBPH (central panel) and anti-EGFP (right panel) antibodies. In the first row it is showed the merge of the acquired signal with the picture of the membrane (marker reference); in the 2nd-4th rows it is showed the signal alone at different time of exposure (software: UVItec Cambridge Alliance). Arrows = specific signal. Lane 1 and 2: S2 cells co-transfected with GAD1 minigene, Actin-GAL4 and Luciferase-RNAi; lanes 3 and 4: S2 cells co-transfected with GAD1 minigene, Actin-GAL4 and TBPH-RNAi; lane 5: S2 cells only.
